# Adaptation and standardization of a Western tool for assessing child development in non-Western low-income context

**DOI:** 10.1186/s12889-016-3288-2

**Published:** 2016-07-28

**Authors:** Teklu Gemechu Abessa, Berhanu Nigussie Worku, Mekitie Wondafrash Kibebew, Jan Valy, Johan Lemmens, Herbert Thijs, Wondwosen Kasahun Yimer, Patrick Kolsteren, Marita Granitzer

**Affiliations:** 1 Department of Psychology, College of Behavioral Sciences and Education, Jimma University, Jimma, Oromia Ethiopia; 2 Department of Population and Family Health, Jimma University, Jimma, Oromia Ethiopia; 3 Department of Healthcare, PXL University College, Hasselt, Belgium; 4 Biocartis NV, Mechelen, Belgium; 5 Department of Epidemiology & Biostatistics, Jimma University, Jimma, Oromia Ethiopia; 6 Department of Food Safety and Food Quality, University of Ghent, Ghent, Belgium; 7 REVAL Rehabilitation Research Center, Biomedical Research Institute, Faculty of Medicine and Life Sciences, University of Hasselt, Hasselt, Belgium

**Keywords:** Adaptation, Child development, Denver II-Jimma, Developmental assessment tool

## Abstract

**Background:**

Due to lack of culturally relevant assessment tools, little is known about children’s developmental profiles in low income settings such as Ethiopia. The objective of this study was to adapt and standardize the Denver II for assessing child development in Jimma Zone, South West Ethiopia.

**Methods:**

Culture-specific test items in Denver II were modified. After translation into two local languages, all test items were piloted and fine-tuned. Using 1597 healthy children 4 days to 70.6 months of age, the 25, 50, 75 and 90 % passing ages were determined for each test item as milestones. Milestones attainment on the adapted version and the Denver II were compared on the 90 % passing age. Reliability of the adapted tool was examined.

**Results:**

A total of 36 (28.8 %) test items, mostly from personal social domain, were adapted. Milestones attainment ages on the two versions differed significantly on 42 (34 %) test items. The adapted tool has an excellent inter-rater on 123 (98 %) items and substantial to excellent test-retest reliability on 119 (91 %) items.

**Conclusions:**

A Western developmental assessment tool can be adapted reliably for use in low-income settings. Age differences in attaining milestones indicate a correct estimation of child development requires a population-specific standard.

## Background

Despite substantial child mortality reduction in Sub-Saharan Africa, many children under-five are still developmentally at risk because of poverty and related risk factors such as malnutrition, poor health and unstimulating home environments [1]. The magnitude of developmental problems is, however, unknown due to lack of culturally relevant tools for assessing development. In the absence of such tools, it is also difficult to correctly determine the developmental effects of interventions targeting children at risk. In rare studies conducted on children at developmental risk, researchers have used tools originally created for technological societies of Europe and North America by either translating or adapting them with little validation [2–6]. Sometimes culture specific test items were totally dropped [7–11] or no adaptation was made [12–14]. Among a Western tool adapted and used worldwide is the American Denver Developmental Screening Test [15] or its revised version, the Denver II [16]. The Denver II is a revised version of the Denver Developmental Screening Test developed in 1967. It was standardized in 1989 on 2,096 American children and published in 1992. It is a screening tool used to identify children between birth and six years who have problems in personal-social (self-help skills and socialization with others), problems in fine motor (eye-hand co-ordination, and manipulation of small objects), problems in language (production of sounds, ability to recognize, understand, and use language), and problems in gross motor (large muscle movements such as sitting, walking, jumping). The Denver II has been used in other countries such as Georgia, Singapore and Sri Lanka by adapting and standardizing it [17–19]. Though it is a simple, quick and feasible to use at institution and home settings to identify children at developmental risks [20], Denver II has not been adapted and validated for use in many low income countries of Africa such as Ethiopia. An indigenous tool similar to it, in style, however, was created for children in Malawi [21]. By using Denver II as a prototype, new test items that were more culturally relevant for Malawian children were created from the Denver Developmental Screening Test, the Denver II and the Griffiths Mental Development Scales.

The main objective of this research, therefore, was to adapt and standardize the Denver II on children between birth and six years of age in the low income context of Jimma Zone of Ethiopia for a more realistic assessment of their development.

## Methods

### Study setting

The study was conducted in Jimma Zone, South West Ethiopia. Within this zone, the population was estimated to be 2.8 million. Jimma Town is the Zonal Capital having about 149, 166 inhabitants [22]. The town is home to more than nine ethnic and linguistic communities communicating mostly in a federal language, Amharic, and a regional language, Afan Oromo. With a mixture of both urban and rural life styles, Jimma town represents the diverse socio-economic, multicultural and multi-lingual Ethiopian society.

#### Adaptation process of the Denver II

The Denver II [16] comprises 125 test items grouped into four domains of child development: 25 personal-social (PS), 29 fine motor (FM), 39 language (LA) and 32 gross motor (GM). These test items are administered using a bell, glass bottle, set of 10 blocks, rattle, pencil, tennis ball, yarn, raisins, cup, white doll, white paper, and baby bottle. Adaptations involved identifying culture specific test items, test objects or materials and then modifying or replacing them to make them culturally relevant. In some cases, instructions for test item administration and criteria of passing were modified.

### Classifying test items under ‘cross-cultural’ and ‘culture-specific’ categories

All test items were first categorized into culture-specific and cross cultural items. Cross cultural relevance of tasks in the test items was assessed using International Classifications of Functions [23]. Culture specific items related to movements (e.g. running, jumping, hopping) were identified using taxonomy of movement skills [24]. Other specific movement skills related to sport, complex movement skills and functional movement skills such as activities of daily living, work, and games are culture-specific. Cross-culturality of items other than movements was assessed using cross cultural psychology [25]. Within this process a local team (psychologists, a special educator and pediatricians) and a Belgian team (child psychiatrist, a pediatrician/ nutritionist, a neuroscientist, a physiotherapist and occupational therapists) worked together.

After translations into the dominant languages (Amharic and Afan Oromo) dialect appropriateness was checked.

### Pilot studies and draft versions

**Fig. 1:**
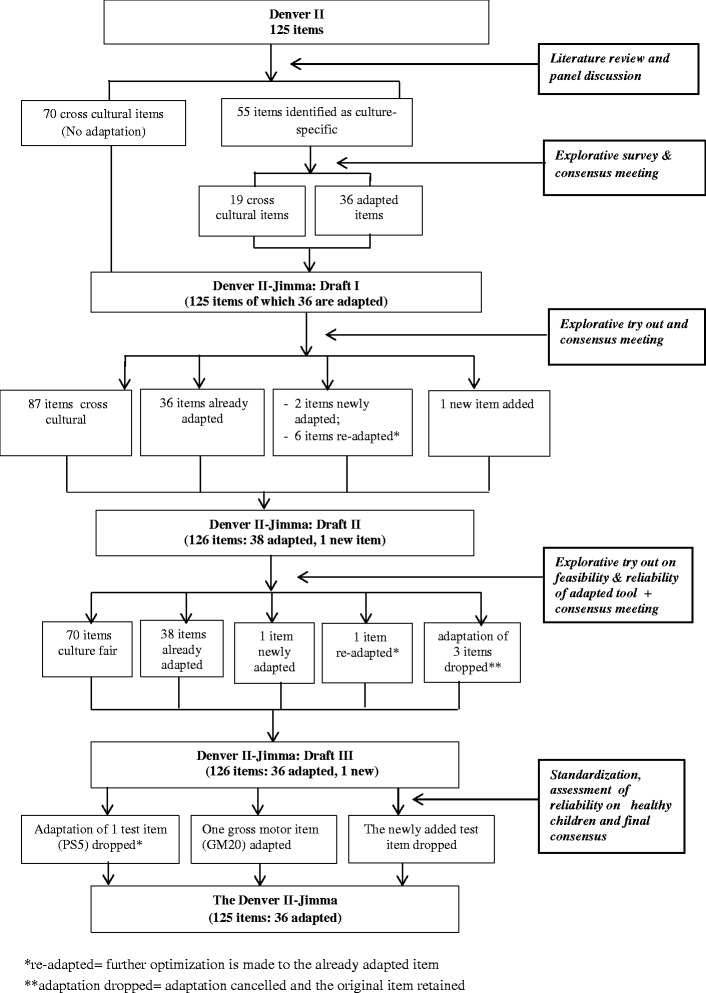
Adaptation and standardization process of the Denver II to Denver II-Jimma

**Fig. 2:**
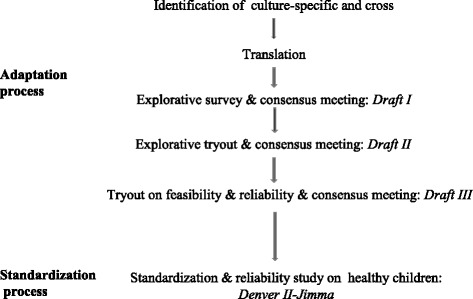
Flow of activities in the adaptation and standardization of the Denver II to Denver II-Jimma

The test items were then piloted on apparently healthy children of accessible parents who consented orally to participate in the study. Draft I emerged based on a survey conducted in 2009 on 19 households. Four urban and 15 rural families were interviewed about the items which were identified as culture-specific (see Fig. 1). Draft I was tried out on eight urban kindergarten children (26–60 months of age; mean = 42.9; SD = ±14.1). Three local study team members, trained in Denver II test item administration, did the testing and the problematic items were discussed at the multidisciplinary team meeting. Re-adaptations resulted in Draft II which was further explored in 2010 for feasibility and reliability on 24 urban kindergarten children (mean age = 51.4 months, SD ± 8.2 months). Testing was conducted by seven trained kindergarten teachers. Further adaptation resulted in Draft III. Figure 2 summarizes the adaptation process.

### Large sample study and standardization

#### Sampling, inclusion and exclusion criteria

Trained nurses collected data using the third draft. Under-six children in Jimma town whose parents could afford to pay preschool education fees were targeted. Such children were assumed to belong to middle or higher socioeconomic level and thus in a context for optimal development. Quota sampling was used to include children in the following age categories (in months): 0–2, 3–8, 9–14, 15–20, 21–26, 27–32, 33–41, 42–53, and 54–65.

Before testing a target child, the mother was interviewed using a 10-point checklist which listed the exclusion criteria. Children, whose mother reported the presence of any of the following potential developmental risks were excluded: prematurely born, birth weight less than 2500 g, very tiny body at birth, instrumentally delivered, or delivered after 24 h of labor, born twins or triplets, born with a chronic health problem, sick during the first year after birth, having observable impairments affecting sight or/and hearing, or/and mobility, having a mother who was seriously sick during pregnancy. Besides, anthropometric measurements were made to assess the nutritional status and exclude malnourished children. Weight was measured with a calibrated digital weighing scale; mid-upper-arm-circumference (MUAC) with a MUAC tape. Anthropometric indices related to length/height were not used for fear of measurement inaccuracy as some children were nervous while positioning them for measurement. Earlier studies have also used weight-for-age to determine child’s nutritional status [7, 26] because the weight-for-age is considered as more comprehensive than the height-for-age [27]. Assessment was done (if the child was well) in the following sequence: developmental assessment, measuring weight, MUAC.

We dispatched questionnaire and study consent form to parents of private kindergarten attending children in Jimma town. The homes of parents who signed the consent form were visited. From 3502 children, only 1682 (mean age = 31.2, SD = 17.75 in months) who were eligible according to the inclusion criteria were tested.. The age of the children ranges from four days to 73.3 months. Initially, 1552 children were tested at home from 11 January to 21 June 2011 and later, 130 children of lower ages (<10 months) were added. Two children of unknown nutritional status and eighty-three malnourished children were excluded during analysis based on weight for age Z-score (WAZ) ≤ −2, or mid-upper-arm-circumference Z-score (MUACZ) ≤ −2 when WAZ was absent.

The study complied with the Helsinki Declaration [28] and was reviewed and approved by Ethical Clearance Board of Jimma University, Ethiopia, and Comite voor Medische Ethiek Universiteit Hasselt, Belgium. Written and oral consents of parents were obtained and children were always tested in the presence of caregivers.

#### Assessment of feasibility and reliability

Feasibility of each test item (meaningfulness of test items, their practicality and ease of administration) was documented during data collection and discussed at final consensus meeting. Inter-rater and test-retest reliabilities were assessed for each test item. Ten female clinical nurses worked in pairs alternately as a tester or an observer. Independent scores were generated for each child by a tester and an observer. These scores by testers and observers were calculated as percentages of agreement to determine the reliability of the test items. Inter-rater reliability was tested on 409 children. Within an average interval of 14 days, 147 of them were tested for test-retest reliability. Inter-rater reliability was not calculated during a re-test condition.

#### Test item administration and scoring system

Test item administration and scoring is the same as in Denver II manual [29]. Each test item on Denver II is presented on a chart by a horizontal bar partitioned into 25, 50, 75 and 90 percentile ages of passing the items. To test a child, his or her age is calculated and a vertical age-line is drawn on the II chart. The testing starts from a test item completely to the left of the age-line. All test items passed by 75 % or more children of same age in the norming sample and by lower ages are counted for a child as *expected passes*. If a child passes three consecutive test items arranged on Denver II test chart, all items to the left are assumed to be passed because they are items achieved at a lower age. These items are called *implied passes*. If a child fails three consecutive test items, it is assumed that all other items arranged to the right on the Denver II chart are failed. These items are *implied failures*. Items passed by a child through testing are *tested passes*. Implied passes and tested passes are added up as *actual passes.* A child’s raw score on each test item is marked as tested pass, implied pass, tested failure, implied failure, refusal, or no opportunity. Categorical and numerical scores were derived for statistical analysis.

##### Categorical score:

For each test item, a binary outcome variable (pass/fail) was created: *pass* (tested pass items) and *fail* (tested failure and refusal). “No opportunity” to perform the item, “implied passes” and “ implied failures” were treated as missing values.

##### Numerical score:

The ratio of *actual passes* to the *expected pass*es was calculated as a *performance ratio* score.

#### Standardization

The objective of the standardization was to determine the ages at which 25, 50, 75 and 90 % of the children pass each of the adapted test items using binary logistic regression.

#### Data management and statistical analysis

Data within the adaptation process (except for reliability) were analyzed qualitatively. Whether or not a test item was culture-specific or cross-cultural was analyzed using theoretical information and discussion among the research team. Data collected during drafting and re-drafting were discussed at interdisciplinary team meetings comprising local and western professionals. Standardization data were entered into EpiData 3.1, double checked, cleaned and exported to SAS 9.3 and STATA 12.1 for analysis. The WAZ and MUACZ scores were calculated as anthropometric indices using WHO Anthro and AnthroPlus and children’s nutritional status determined against WHO reference standard [30].

Predicted ages at which 25, 50, 75 and 90 % of the norming sample passed each test item were derived from the models and calculated as milestone ages. Using the categorical score “pass/fail”, binary logistic regression model was fitted for each test item by entering child age in days as a single covariate. Predicted probabilities of passing were calculated from alpha and beta coefficients. Goodness of the fit was assessed using Hosmer and Lemeshow test statistic at 5 % level of significance. Items with poor model fit (*p*-value <0.05) were refitted using cubic splines [31].

Age of attaining milestones by Denver II and Denver II-Jimma norming samples were compared on 90 percentile age. More than 10 % difference was considered clinically significant.

Reliability was assessed at item and domain levels. Item reliability was calculated as a percentage of agreement between a tester score and an observer score (inter-rater), and between the first test and retest scores (test-retest) for the same child. Chance agreements were corrected using Cohen’s kappa. Kappa values by Landis and Koch [32] were used for interpretation: value below 0.20 as slight; between 0.21 and 0.40 as fair; between 0.41 and 0.60 as moderate, between 0.61 and 0.80 as substantial, and between 0.81–1.00 as excellent agreement. Where kappa could not be calculated, percentages of agreement for events were determined: 70 % or higher was considered as acceptable.

Domain reliability was evaluated using intra-class correlation coefficients. First, performance ratio scores were generated for each of the four domains separately. Then, the correlations between tester and observer performance ratio scores at two testing moments (test and retest) were computed for each domain as inter-rater and test-retest intra-class correlation.

## Results

### Outcome of the adaptation

Of the 125 Denver II test items, 55 (20 PS, 18 FM, 15 LA, 2 GM) were theoretically identified as culture-specific. These 55 items were piloted through exploratory survey and discussed at a consensus meeting. Only 36 of them needed adaptation. The other 19 items were retained as was in the original (Fig. 1). A tryout revealed difficulties with eight (6 LA and 2 PS) test items. Further fine-tuning resulted in Draft II (36 adapted, 1 newly added, 89 original Denver II items). Inter-rater reliability of Draft II was excellent (kappa > 0.83) for all tested items. For items with skewed data distribution kappa could not be computed. Their percentages of agreement, however, were all acceptable (71.4 to 95.2).

Some test items were found practically difficult to administer or still difficult for children to understand even after initial adaptation. Hence, to make sure that test items were feasible to administer, understandable for children and caregivers, further adaptations were made. One item from PS was adapted, and another re-adapted; and the adaptations of three LA items were dropped. This resulted in the Denver II-Jimma-Draft III, which comprises 36 adapted (18 personal social, 10 fine motor, 8 language), 1 newly added (toilet going), and 89 original Denver II items.

At the final consensus meeting following the standardization study, one gross motor (walk up steps) was adapted, the newly added item was dropped, and adaptation of one personal social item was dropped. This resulted in the final Denver II-Jimma having 36 adapted items (Table 1).

**Table 1:** Descriptions of adaptation made to the Denver II test items to make Denver II- Jimma

Denver II test items adapted	Item code	Description of adaptation of the item
*Work for toy* ^a^	*PS5*	*Rattle or culturally used tools such as small pebbles “ calle” or “ elela” were selected.*
Feed self	PS6	The items "cracker", "cookie" or “any finger food” were replaced by locally used food such a piece of "bread", "cake", "biscuit", "injera" or a piece of sugar-cane.
Play pat-a-cake	PS7	Replaced by "play a clapping game": a culturally equivalent game played by clapping hands.
Wave bye-bye	PS9	Modified Cultural difference in expressing goodbye: "saying" or "waving" goodbye
Imitate (household) activities	PS11	Activities such as "vacuuming", or "talking on the phone" were replaced by activities such as ...."cooking" or "washing clothes".
Drink from cup	PS12	….child can hold a regular cup or glass and drink from it without help replaced by a regular "cup or glass" or any suitable container used in the family.
Use spoon/fork	PS14	Modified as "eat using hand or sppon/fork. The child uses a spoon or fork to eat. is modified as “the child is able to eat independently by using his/her fingers or a spoon or fork”
Remove garment	PS15	… items such as…."jackets", "pants", are modified as… items such as "blouse", "dress" or "trousers"
Feed doll	PS16	The criterion to pass the test item “ if the child places the bottle to the doll's mouth, or tries to put it to the mouth"… is modified as “if the child imitates putting food into the doll’s mouth or if the child imitates breast feeding”. The use of bottle feeding is being discouraged and many mothers do not practice. Since toy bottle was found strange for many children, performing the task is not expected to pass the test item .
Put on clothing	PS17	….clothing, such as "underpants", "socks" are modified as … clothing, such as "blouse", "trousers", "dress", "skirt"
Brush teeth with help	PS18	Replaced by "wash mouth with help"… if the child "brushes his/her teeth with some help"…is modified as if the child "washes his/her mouth with some help"
Wash and dry hands	PS19	Criteria for passing the test item is modified as … child can "wash both sides of hands properly" (because hands are culturally dried by dripping water off the hands). Use of soap and towels are not required to pass the item.
Name friend	PS20	Replaced by "name playmate"
Put on T-shirt	PS21	"pullover" …is replaced by "T-shirt" or "blouse" …
Dress, no help	PS22	First: "at least play-clothes" …is modified as …"his/her own clothes", because children may not have many alternative clothing. Finally: adaptation dropped
Play board/card games	PS23	Modified as "play social games" …. "board" or "card" games, … “Candy Land” or “Old Maid”… is modified as “joins in simple "group games", like "hide and seek"
Brush teeth, no help	PS24	"brush teeth, no help" replaced by "clean face, no help". … if the child "washes his/her own teeth" …is replaced by… if the child "washes and dries" his/her face (eyes, nose, mouth and teeth) …. this is usually done by using water and fingers)
Prepare Cereal	PS25	First: … if the child can prepare a bowl of cereal …… is modified as …if the child can prepare his/her own breakfast, including taking bread (or injera,…) from the shelf, taking a cup and pouring a liquid (water, milk, juice) in it.. Finally: modified as "serve oneself 'injera'" (cultural food served with stew)
*Toilet going* ^b^	PS26	First: Toilet going (new item): Ask the caregiver if the child can independently use latrine or other facilities available for the family. PASS if they report the child can independently use toilet or latrine or available facility Finally: item dropped
Regard raisin	FM6	Is replaced by "Regard coffee bean".
Rake raisin	FM9	Is replaced by "Rake coffee bean"
Thumb-finger grasp	FM12	… "raisin" which is used as an object for child to grasp is replaced by "coffee bean"
Scribbles	FM15	The instruction …”do not show him/her how to scribble”… is modified as.… you may write your name using the pencil to let the child who have never ever seen a pencil before that it is something to write with
Dump raisin, demonstrated	FM16	Replaced by "dump coffee bean, demonstrated"
Copy 0	FM23	The instruction “ you may show how to hold the pencil” is added to familiarize a child who has never seen a pencil before
Draw person – 3 parts, 6 parts	FM24 & FM 28	The instruction… “You may show how to hold the pencil”… is added to familiarize a child who has never seen a pencil before
Copy +	FM25	The instruction… “You may show how to hold the pencil”… is added familiarize a child who has never seen a pencil before
Copy □	FM29	The instruction… “You may show how to hold the pencil”… is added to familiarize a child who has never seen a pencil before
Body parts – 6	LA21	A testing object "white doll" replaced by a black or chocolate colour doll. The use of either a white doll or a chocolate colour doll based on need of child was suggested based on repeated observation.
Name colours – 1,4	LA27 & LA34	First: Criterion for passing test item modified as, “ Child could pass the test if he/she refers to an object with the same colour: sky/water for blue, grass/tree for green, sun for yellow and blood for red. Finally: The "blue" and "green" colour cubes are replaced by "black" and "white" colour cubes.
Use of objects – 2, 3	LA28 & LA 30	....“What is a 'pencil' used for?”…. is replaced by ….“What is a 'bed' used for?”….
Define words – 5, 7	LA35 & LA39	The words "desk", "curtain", "lake", are replaced by the words "knife", "firewood", "river"
Opposites – 2	LA37	“If 'fire' is 'hot', 'ice' is..." was replaced by "if a stone is heavy, a feather/leaf is …..”
Walk up step^c^	GM20	Walk up steps where there are steps in homes, and/or walk up-ward on steep location/ climbs and passes over an elevated door-step

^a^The adaptation of this test item was dropped at a final consensus meeting

^b^Newly added test item removed at final consensus meeting

^c^The adaptation of this test item was added at a final consensus meeting

### Outcome of the standardization

#### Characteristics of the standardization sample

Nearly equal number of boys and girls participated in the study. About 95 % of the caregivers rated themselves as belonging to middle or higher socio-economic standard.

The Oromo, as the largest ethnic group, seem to have been fairly represented (45.1 %). Only 9.3 % of mothers of children enrolled in the study are illiterate (Table 2).

**Table 2:** Characteristics of study participants

Characteristics	No. (%)	Characteristics	No. (%)
Children’s (n = 1682)		Mother’s (n = 1588)	
Sex		Ethnicity	
Male	833 (49.5)	Oromo	716 (45.1)
Female	849 (50.5)	Amhara	363 (22.9)
Nutritional status		Tigre	46 (2.9)
Normal range^a^		Gurage	140 (8.8)
Male	789 (46.9)	Dawuro	148 (9.3)
Female	808 (48)	Keficho	72 (4.5)
Malnourished^b^		Wolaita	30 (1.9)
Male	43 (2.6)	Others	62 (3.9)
Female	40 (2.4)	Missing/Unknown	11 (0.7)
Unknown status		Perceived socio-economic status	
Male	1 (0.06)	High	58 (3.7)
Female	1 (0.06)	Middle	1443 (90.9)
		Low	65 (4.1)
Mother’s (n = 1588)		Very low	0
Education level		Missing/unknown	22 (1.4)
Illiterate	147 (9.3)		
Grades 1–8	546 (34.4)	Religion	
Grades 9–12	485 (30.5)	Islam	558 (35.1)
Certificate after grade 12	98 (6.2)	Orthodox Christian	725 (45.7)
Diploma	247 (15.6)	Protestant	271 (17.1)
Degree and above	55 (3.5)	Catholic	23 (1.4)
Missing/unknown	10 (0.6)	Others	6 (0.4)
		Missing	5 (0.3)

^a^(WAZ > −2 where both WAZ and MUACZ score are present; and MUACZ > −2 where WAZ score is missing); ^b^(WAZ ≤ −2 where both WAZ and MUACZ score are present; and MUACZ ≤ −2 where WAZ score is missing)

*WAZ* Weight-for-age-Z-score, *MUACZ* Mid-upper-arm-circumference Z score

#### The Denver II-Jimma Age Milestones

**O**f the 126 test items separately fitted on logistic model, 66 items fitted well. Three items (PS1, LA1, GM1) could not be fitted because all tested children passed them. Fifty-seven items showed poor fit (13 PS, 18 FM, 11 LA, 15 GM). The model fitness for 39 of these were improved by refitting using cubic splines. On lots of test items, the Denver II-Jimma differed from Denver II on 50, 75 and 90 % ages of attaining milestones (Table 3).

**Table 3:** The Denver II-Jimma with its age norms (in months) for 25, 50, 75 and 90 % of children passing the test items within the different domains

Item code	Item label	25 %	50 %	75 %	90 %	Item code	Item label	25 %	50 %	75 %	90 %
	Personal social domain						Fine motor domain				
PS1	Regard face	birth^b^	birth^b,d^	birth^b,d^	birth^b,d^	FM1	Follow to midline	birth^b^	0.1^f^	0.1^e^	0.2^e^
PS2	Smile responsively	0.8	1.1^f^	1.3^d^	1.6^d^	FM2	Follow past midline	1.3	1.5^f^	2.2^e^	2.7^d^
PS3	Smile spontaneously	1.1	1.4^e^	1.6^f^	1.9^e^	FM3	Grasp rattle	2.4	3.0^d^	3.6^d^	4.1^d^
PS4	Regard own hand	1.6	1.,8^e^	3.2^e^	4.1^d^	FM4	Hands together	2.5	3.1^f^	3.7^f^	4.3^d^
PS5	Work for toy	3.1	4.5^d^	6.0^f^	7.6^f^	FM5	Follow 180°	3.1	3.7^f^	4.3^f^	4.9^d^
PS6	**Feed self** ^a^	4.0	5.9^d^	8.2^f^	10.5^f^	FM6	**Regard coffee bean** ^a^	4.1	4.5^f^	5.0^f^	5.4^d^
PS7	**Play clapping game** ^a^	6.9	8.1^e^	9.4^d^	10.6^d^	FM7	Reaches	4.2	4.7^d^	5.2^d^	5.6^d^
PS8	Indicate wants	6.1	8.1^e^	10.1^d^	12.2^d^	FM8	Look for yarn	4.9	5.4^d^	6.0^d^	6.6^d^
PS9	**Wave bye-bye/Say good-bye** ^a^	8.2	10.0^f^	11.8^f^	13.6^d^	FM9	**Rake coffee bean** ^a^	5.3	5.9^d^	6.4^d^	6.9^d^
PS10	Play ball with examiner	10.4	12.,2^f^	14.0^f^	15.8^d^	FM10	Pass cube	6.2	7.4^f^	8.5^f^	9.7^f^
PS11	**Imitate activities** ^a^	9.7	11.6^d^	13.4^d^	15.2^d^	FM11	Take 2 cubes	5.8	6.9^f^	8.2^f^	9.5^d^
PS12	**Drink from cup or glass** ^a^	9.7	11.8^d^	13.9^d^	16.0^d^	FM12	**Thumb-finger grasp** ^a^	6.5	7.5^d^	8.7^d^	9.9
PS13	Help in house	12.9	15.9^f^	18.4^f^	20.9^f^	FM13	Bang 2 cubes held in hands	7.4	9.3^f^	11.3^f^	13.3^f^
PS14	**Eats using spoon/fork/fingers** ^a^	11.7	14.5^d^	17.2^d^	20.0	FM14	Put block in cup	8.7	10.2^d^	11.7^d^	13.1^d^
PS15	**Remove garment** ^a^	15.5	20.4^f^	24.5^f^	28.6^f^	FM15	**Scribbles** ^a^	10.7	13.4^d^	16.0^d^	18.6^f^
PS16	**Feed doll** ^a^	14.4	19.5^f^	24.5^f^	29.7^f^	FM16	**Dump coffee bean, demonstrated** ^a^	10.9	13.5^d^	16.0^d^	18.5^d^
PS17	**Put on clothing** ^a^	25.3	31.8^f^	38.1^f^	44.5^f^	FM17	Tower of 2 cubes	12.6	15.6^d^	18.3^d^	21.0^d^
PS18	**Wash mouth with help** ^a^	20.4	24.8^f^	29.1^d^	33.4^d^	FM18	Tower of 4 cubes	16.4	19.3^d^	22.2^d^	25.1^d^
PS19	**Wash and dry hands** ^a^	22.9	27.4^f^	32.4^f^	37.4^d^	FM19	Tower of 6 cubes	19.7	23.1^d^	26.6^d^	30.1^d^
PS20	**Name playmate** ^a^	22.8	28,3^d^	33.4^d^	38.6^d^	FM20	Imitate vertical line	25.9	32.4^f^	37.7^f^	43.0^f^
PS21	**Put on t-shirt** ^a^	34.2	40.8^f^	47.3^f^	53.8^f^	FM21	Tower of 8 cubes	22.1	29.4^f^	35.0^d^	40.6^d^
PS22	**Dress, no help** ^a^	43.9	50.5^f^	57.0^f^	63.5^f^	FM22	Thumb wiggle	27.0	32.8^d^	38.4^d^	44.2^d^
PS23	**Play social games** ^a^	31.8	41.6^f^	51.2^d^	60.9^d^	FM23	**Copy** ^a^ **O**	38.7	43.1^d^	47.3^d^	51.6^d^
PS24	**Clean face, no help** ^a^	37.9	46.8^f^	55.5^d^	64.4^d^	FM24	**Draw person—3 parts** ^a^	44.0	48.2^d^	52.3^d^	56.4^d^
PS25	**Serve oneself** ***injera*** ^a^	31.7	46.5^d^	61.2^f^	76.0^d^	FM25	Copy^a^ +	33.8	40.4^d^	46.9^d^	53.4^d^
PS26	Toilet-going^c^	60.1	70.4^d^	80.6^d^	90.8^d^	FM26	Pick longer line	33.8	40.4^d^	46.9^d^	53.4^e^
						FM27	Copy □ demonstrated	45.7	51.8^d^	57.7^d^	63.6^d^
						FM28	Draw person—6 parts^a^	52.4	57.5^d^	62.4^d^	67.4^d^
						FM29	Copy^a^□	52.3	58.2^d^	64.0^d^	69.8^d^
	Language domain						Gross motor domain				
LA1	Respond to bell	birth^b^	birth^b,d^	birth^b,d^	birth^b,d^	GM1	Equal movement	birth^b^	birth^b,d^	birth^b,d^	birth^b,d^
LA2	Vocalizes	birth^b^	birth^b,d^	0.3^f^	1.0^f^	GM2	Lift head	birth^b^	0.1^f^	0.4^f^	0.7^f^
LA3	“Ooo”/ Aah	1.4	1.5^f^	1.6^d^	1.6^e^	GM3	Head up 45°	1.6	2.4^f^	2.3^f^	2.6^d^
LA4	Laugh	2.0	2.2^f^	2.5^d^	2.7^e^	GM4	Head up 90°	2.9	3.2^f^	3.6^f^	3.9^d^
LA5	Squeals	2.2	2.5^f^	2.8^d^	3.2^e^	GM5	Sit head steady	3.0	3.3^f^	3.6^f^	3.9^d^
LA6	Turn to rattling sound	3.7	4.3^f^	4.8^d^	5.4^d^	GM6	Bear weight on legs	3.1	3.4^f^	3.6^d^	3.8^e^
LA7	Turn to voice	4.5	5.2^f^	5.8^d^	6.5^d^	GM7	Chest up-arm support	3.9	4.3^f^	4.6^f^	4.9^d^
LA8	Single syllables	4.6	5.5^d^	6.3^d^	7.1^d^	GM8	Roll over	3.8	4.4^f^	4.9^f^	5.4^d^
LA9	Imitate speech sounds	5.2	6.5^f^	7.8^f^	9.1^d^	GM9	Pull to sit, no head lag	4,4	4.9^f^	5.5^f^	6.0^d^
LA10	Dada/Baba/Mama, non-specific	5.7	6.7^d^	7.8^d^	8.8^d^	GM10	Sit, no support	5,4	6.0^d^	6.6^d^	7.3^d^
LA11	Combine syllables	6.2	7.5^f^	8.8^f^	10.1^d^	GM11	Stand, holding on	6,5	7.3^d^	8.3^d^	9.3^d^
LA12	Jabbers	7.0	8.5^f^	10.0^f^	11.5^d^	GM12	Pull to stand	7,2	8.0^d^	8.9^d^	9.9^d^
LA13	Dada/Mama/baba, specific	8.8	10.2^f^	11.6^d^	12.9^d^	GM13	Get to sitting	7,4	8.3^d^	9.2^d^	10.1^d^
LA14	One word	10.1	11.9^d^	13.6^d^	15.3^d^	GM14	Stand 2 s	8,6	9.7^d^	10.7^d^	11.6^d^
LA15	2 words	11.5	13.8^f^	15.9^d^	18.0^d^	GM15	Stand alone	9,8	11.3^d^	12.3^d^	13.4^d^
LA16	3 words	13,5	15.6^f^	17.7^f^	19.8^d^	GM16	Stoop and recover	11,6	13.2^d^	14.8^f^	16.4^f^
LA17	6 words	16,5	19.1^f^	21.8^f^	24.4^f^	GM17	Walk well	11,3	13.1^d^	14.9^f^	16.7^f^
LA18	Point 2 pictures	20,8	24.0^f^	27.2^f^	30.3^f^	GM18	Walk backwards	11,9	14.9^d^	17.4^f^	19.9^f^
LA19	Combine words	18,7	21.3^d^	23.9^d^	26.4^d^	GM19	Runs	14,4	16.6^d^	18.9^d^	21.2^d^
LA20	Name 1 picture	20,1	23.2^f^	26.2^f^	29.3^d^	GM20	**Walk up steps** ^a^	13,9	16.9^d^	19.6^d^	22.3^d^
LA21	**Body parts 6** ^a^	20,3	23.0^f^	25.6^f^	28.2^d^	GM21	Kick ball forward	14,2	17.6^d^	21.0^d^	24.4^d^
LA22	Point 4 pictures	25,9	31.0^f^	35.9^f^	40.9^f^	GM22	Jump up	24.0	27.0^f^	31.0^f^	35.0^f^
LA23	Speech, half understandable	20,5	24.2^f^	27.8^d^	31.5^e^	GM23	Throw ball overhand	16.9	22.1^d^	27.2^f^	32.4^d^
LA24	Name 4 pictures	27,6	32.5^f^	37.3^f^	42.1^f^	GM24	Broad jump	31.7	35.6^d^	39.3^f^	43.1^f^
LA25	Know 2 actions	24,5	29.5^d^	34.4^d^	39.4^d^	GM25	Balance each foot 1 s	23.4	28.9^d^	33.0^d^	37.1^d^
LA26	Know 2 adjectives	30,9	35.1^d^	39.1^d^	43.3^d^	GM26	Balance each foot 2 s	23.9	31.2^e^	36.4^e^	41.6^e^
LA27	**Name 1 color** ^a^	40,3	45.1^d^	49.8^f^	54.6^f^	GM27	Hops	31.9	38.5^d^	45.0^d^	51.5^d^
LA28	**Use of 2 objects** ^a^	30,4	35.6^f^	40.7^d^	45.8^d^	GM28	Balance each foot 3 s	29.8	36.1^d^	42.2^e^	48.4^e^
LA29	Count 1 block	36,6	41.9^d^	47.0^f^	52.2^f^	GM29	Balance each foot 4 s	33.8	40.0^e^	46.0^e^	52.1^e^
LA30	**Use of 3 objects** ^a^	32,3	37.5^d^	42.7^d^	47.8^d^	GM30	Balance each foot 5 s	39.2	44.8^e^	50.2^e^	55.8^e^
LA31	Know 4 actions	32,4	38.5^e^	44.4^f^	50.4^d^	GM31	Heel-to-toe walk	49.1	55.5^d^	61.8^d^	68.1^d^
LA32	Speech all understandable	28,4	36.3^f^	44.0^f^	51.8^d^	GM32	Balance each foot 6 s	41.8	47.9^e^	53.8^e^	59.7^e^
LA33	Understand 4 prepositions	30,8	37.5^d^	44.0^d^	50.7^e^						
LA34	**Name 4 colors** ^a^	51,9	56.8^f^	61.5^f^	66.3^f^						
LA35	**Define 5 words** ^a^	47,4	54.3^f^	61.0^d^	67.8^d^						
LA36	Know 3 adjectives	34,5	40.6^d^	46.5^d^	52.5^e^						
LA37	**Count 5 blocks** ^a^	49,0	54.3^d^	59.4^d^	64.6^d^						
LA38	Opposites-2	48,5	54.0^d^	59.2^d^	64.5^d^						
LA39	**Define 7 words** ^a^	59,1	66.8^f^	74.2^f^	81.8^f^						

*PS* personal social, *FM* fine motor-adaptive, *LA* language, *GM* gross motor items

^a^Adapted test items (written in bold)

^b^The child is able to perform or pass the task soon after birth

^c^Newly added test item removed at final consensus meeting

^d^Item achieved at no significantly different ages it is achieved in Denver II (achieved at similar age)

^e^Item achieved at a significantly earlier age than the age it is achieved in Denver II (achieved at earlier age)

^f^Item achieved at a significantly later age than the age it is achieved in Denver II (achieved at later age)

The 90 % age of milestones attainment on Denver II-Jimma significantly differed from Denver II on 42 (33.6 %) items (9 PS, 6 FM, 15 LA, and 12 GM). Fifteen test items were attained at an earlier age and 27 items at a later age than they are achieved on the Denver II. The remaining 83 (66.4 %) milestones were achieved at a similar age (Table 3).

#### Reliability of the Denver II-Jimma

Table 4 summarizes the results for the reliability of the Denver II-Jimma at individual test item and overall domain levels. Inter-rater reliability was excellent except for two test items which showed substantial agreement: (“PS5: work for toy”, kappa = 0.74 and FM5: “follow 180 degrees”, kappa = 0.78). Majority (above 90 %) of the test items have a substantial to excellent test-retest reliability. Only one test item (FM 8: “look for yarn”, kappa = 0.33) showed unacceptable kappa values. The Denver II-Jimma also demonstrated very high intra-class correlations on all domains of development (Table 4).

**Table 4:** Reliability of Denver II-Jimma at item level indicated by inter-rater^a^ and test re- test^b^ kappa values, and at domain-level indicated by inter-rater and test-retest intraclass correlation coefficients

Reliability Measures	PS (26 items)	FM (29 items)	LA (39 items)	GM (32 items)	Total (126 items)
***Inter-rater*** (kappa values)					
Excellent (0.81–1.00)	21 (80.8 %)	24 (82.8 %)	34 (87.2 %)	27 (84.4 %)	124 (98.4 %)
	4 (15.4 %)***	4(13.8 %)***	5(12.8 %)***	5(15.6 %)***	
Substantial (0.61–0.80)	1(3.8 %)	1 (3.4 %)	-	-	2 (1.6 %)
Acceptable (0.41–0.60)	-	-	-	-	-
Poor (<0.41)	-	-	-	-	-
***Inter-rater*** (ICC)^C^, [95 % CI]	0.983, [0.979-0.986]	0.982, [0.978-0.985]	0.951, [0.940-0.959]	0.967, [0.961-0.973]	
***Test retest*** (kappa values)					
Excellent (0.81–1.00)	15 (57.7 %)	4(13.8 %)	14 (35.9 %)	14 (43.8 %)	69 (54.8 %)
	4 (15.4 %)***	5 (17.24 %)***	6 (15.4 %)***	5 (15.6 %)***	
		1(3.45)*	1(2.6 %)**		
Substantial (0.61–0.80)	5 (19.2 %)	13 (44.8 %)	16 (41 %)	11 (34.4 %)	45 (35.7 %)
Acceptable (0.41–0.60)	2 (7.7 %)	5 (17.24 %)	2 (5.1 %)	2 (6.3 %)	11 (8.7 %)
Poor (<0.41)		1 (3.45 %)	-	-	1 (0.8 %)
Test-retest (ICC)^d^, [95 % CI]	0.802, [0.721–0.859]	0.831, [0.736–0.888]	0.840, [0.773–0.887]	0.793, [0.711–0.852]	

*PS* Personal social, *FM* Fine motor-adaptive, *LA* Language, *GM* Gross motor, *ICC* intraclass correlation coefficient, *CI* confidence interval

^***^Kappa value not calculated but percentage of agreement is 100

^**^Kappa value not calculated but percentage of agreement is 93.3

^*^Kappa value not calculated but percentage of agreement is 90.91

^a^Agreement between two measurements done independently at a time

^b^Agreement between measurements repeated at a different time

^c^One-way random effect model is used and shows very high correlation

^d^Two-way random effect model is used and shows high correlation

#### Final consensus on Denver II-Jimma

As bottle feeding is being discouraged in line with WHO’s recommendation, it is agreed that the test item “Feed doll” should be administered without using a toy bottle. Local material “*Callee*” initially suggested to replace the object “rattle” for administering the item “work for toy” was so risky for babies because it is small and could be swallowed. Hence, the adaptation was dropped. A newly added test item (“toilet going”) was found difficult to perform before the age of six years and was thus eliminated. A gross motor item “Walk up steps” was not possible to assess in homes lacking steps. In such cases, care givers were asked if a child is able to walk up-ward a steep position or cross elevated doorstep. Hence, the Denver II-Jimma finally evolved as a-125-test item tool with 36 (28.8 %) adapted test items: 17 PS, 10 FM, 8 LA and one GM items.

## Discussion

In order to provide early intervention for children developmentally at risk, correct assessment of their developmental status is an essential first step. Since development is influenced by the sociocultural contexts, instrument assessing child development should take culture into account. The tools should also be psychometrically valid. While child development tools created in western cultural contexts are psychometrically valid, they may not be culturally relevant to use with African children. Many agree that culturally relevant developmental assessment tools should be either created [33] or adapted from tools developed in other cultures [5, 34]. Adapting an existing tool is less expensive and more suitable to maintain construct validity of a tool across different settings.

In this study the Denver II created in the Western socio-cultural context, was adapted and standardized on Ethiopian children in Jimma town. The Denver II-Jimma evolved as a culturally relevant tool, ready to use for children from birth to six years in the multicultural and multilingual communities in the Jimma Zone, south west of Ethiopia. In the adaptation process, 36 items of the 125 in the Denver II test were modified. No test item was dropped, and this would guarantee to maintain the objectives and content validity of the original tool. Content validation was conducted by going through each test item at different meetings by the multidisciplinary research team with knowledge of local and western cultures. First, the objective of testing each Denver II test item, specific skill or competence assessed was discussed. Then the equivalence of the adapted version of the test item with the original one was examined in line with the objective, skill or competence assessed. This process was meant to maintain both content and construct validity.

Adaptation was predominantly in personal social test items. Only one gross motor item was adapted. This is consistent with other studies [19, 34]. Personal social skills seem to be more prone to socio-cultural influences than gross motor skills.

Feasibility and reliability of all test items were ensured during the adaptation process through piloting and fine-tuning. Good inter-rater and test-retest reliabilities were demonstrated during testing at schools by kindergarten teachers, and, at home by clinical nurses indicating that the Denver II-Jimma is reliable to use at different settings by different professionals. A strong intra-class correlation across all the domains also shows good overall reliability. Similar to the Denver II [16], inter-rater reliability seems to be better than the test-retest reliability.

Milestones attainment on Denver II and Denver II-Jimma were compared on 90 percentile ages. Though there is no significant difference on majority (66.4 %) of the test items, a clinically significant difference was observed on 42 items. Such a difference was also reported in earlier studies [17–19, 34–39]. The difference was found for both the culture specific and the cross-cultural items. This finding of achieving milestones at different ages seems to justify the need to have separate normative standards for valid interpretation of test results from different socio-cultural contexts.

Differences are observed in the number of Denver II test items adapted in different settings. While 36 test items are adapted in the present study, only two items (personal social item “’play-pat-a-cake’ and language item Baba or Mama, nonspecific’) were modified while standardizing and adapting Denver test to Tbilisi [40] children in Georgia. Only five test items (4 personal social and one language) were modified while adapting and standardizing Denver II on Sri Lankan children [19]. In Singapore, 77 Denver II items (67 %) were shared with the adapted and standardized Singaporese version [17]. Such findings seem to show that the number of test items needing adaptation varies in different socio-cultural contexts.

There are also differences in ages of attaining milestones in different settings. With a difference of more than 10 % on 90 percentile passing age, the Singapore differed on more than 30 items (20.1 %); the Denver–Tbilisi on 25 items (24 %), the Denver II-Jimma on 42 items (33.6 %) with the original Denver II. A comparison of the Sri Lankan norm with the Singapore and the Denver II norms also showed a difference of more than one month in ages of attaining milestones in more than 75 % of items in all domains [19]. The differences in ages of attaining milestones in the present study produced findings that are expected and consistent to earlier studies.

Taking in account that the Denver II-Jimma should be an ‘ideal reference’ to detect children at developmental risk, and monitor the general recovery of the child during rehabilitation, much care was spent on the standardization. Standardization therefore was done on a large sample of healthy children by excluding those with obvious disabilities and at risk during pre and perinatal stages of development. Children from comparatively very low-income families were not included for fear that such children are at higher developmental risks related to malnutrition and developmentally non-stimulating home environment. Moreover, significantly malnourished children were also excluded from the analysis since malnutrition affects development.

An important aspect of the adaptation process is the involvement of an interdisciplinary team comprising academicians and practitioners from both the western and the local cultures. They were found instrumental in understanding both contexts while making relevant adaptations. Such a team composition was either not reported or considered in other similar studies.

The study is not also without limitations. First, though the Denver II is valid and is still in use in the western world, it was standardized 24 years ago. This standard is, however, still in use. Therefore, this study compared the data from two different time points. Second, though it is claimed that adaptation improves sensitivity [40], the Denver II-Jimma could still be a subject of limitation of the Denver II: weak specificity [41]. With adaptation of the traditional scoring and interpretation, however, the Denver II is regarded as more suitable for children with medically complex conditions [42], and a valid tool, particularly in assessing the language and fine motor skills of children with neurodevelopment risks [43].

## Conclusion

This study demonstrated how a Western tool can be effectively adapted to a non-Western setting. With high inter-rater and test retest reliability, the Denver II-Jimma quickly assesses development of under six children, and is easy to use by first-line health workers and kindergarten teachers at home, school or health centers. Difference in milestones achievement ages on the adapted tool and on its originating Western tool shows that creating a local standard using the adapted tool is necessary for a valid interpretation of results. The study was conducted on children of diverse cultural, linguistic and ethnic communities. Hence, the result could be generalized to many other populations of Ethiopian children. However, some minor modifications may be needed in certain contexts which significantly differ from the present study setting. Future research has to examine if the tool can be used in other similar settings.

## Abbreviations

FM, gross motor; GM, gross motor; LA, language; MUAC, Mid-upper circumference; MUACZ, Mid-upper arm circumference z score; PS, personal social; WAZ, weight-for-age z score.
